# Erythropoietin ameliorates diabetes-associated cognitive dysfunction *in vitro* and *in vivo*

**DOI:** 10.1038/s41598-017-03137-6

**Published:** 2017-06-05

**Authors:** Meng Wang, Wenhui Yan, Yuan Liu, Hao Hu, Qiang Sun, Xinlin Chen, Weijin Zang, Lina Chen

**Affiliations:** 1 Department of Pharmacology, School of Basic Medical Sciences, Xi’an Jiaotong University Health Science Center, Xi’an, 710061 Shaanxi China; 2 Key Laboratory of Environment and Genes Related to Diseases (Xi’an Jiaotong University), Ministry of Education, Xi’an, 710061 Shaanxi China; 3 Shijiazhuang Obstetrics and Gynecology Hospital, Shijiazhuang, 050000 Hebei China; 4 Institute of Neurobiology, School of Basic Medical Sciences, Xi’an Jiaotong University Health Science Center, Xi’an, 710061 Shaanxi China

## Abstract

Several studies indicate that erythropoietin (EPO) has remarkable neuroprotective effects in various central nervous system disorders, while little is known about the effects of EPO in diabetes-associated cognitive dysfunction. Therefore, the present study aimed to investigate whether EPO ameliorates diabetes-associated cognitive dysfunction *in vivo* and *in vitro*. We investigated the protective effects of EPO on high-glucose (HG)-induced PC12 cell death and oxidative stress. The effects of EPO (300 U/kg administered three times a week for 4 weeks) on diabetes-associated cognitive decline were investigated in diabetic rats. EPO significantly increased cell viability, increased the activity of superoxide dismutase, decreased the production of malondialdehyde and reactive oxygen species, and decreased the apoptosis rate. Additionally, LY294002, a phosphatidylinositol 3-kinase (PI3K) inhibitor, abolished the protective effects of EPO in HG-treated PC12 cells. In diabetic rats, EPO prevented deficits in spatial learning and memory in the Morris water maze test. The results of real-time PCR and Western blotting showed that EPO upregulated EPO receptor, PI3K, and phosphorylated Akt2 relative to unphosphorylated Akt2 (p-Akt2/Akt2) and downregulated glycogen synthase kinase-3β (GSK-3β). These studies demonstrate that EPO is an effective neuroprotective agent in the context of diabetes-associated cognitive dysfunction and show that this effect involves the PI3K/Akt/GSK-3β pathway.

## Introduction

With the increase of the world’s aging population and the extension of the average life span, metabolic and neurodegenerative diseases, which are related to age, have become a global problem. The prevalence of diabetes mellitus (DM) and Alzheimer’s disease (AD) is increasing, and these diseases seriously affect people’s quality of life. Neurons are extremely metabolically active cells and have high demands for energy to support their biological activity. Therefore, neuronal function and survival are highly affected by changes in brain glucose metabolism^1^. The main pathological characteristics of type 2 DM are hyperglycemia and insulin resistance (IR), both of which have a harmful effect on cognitive function and induce diabetes-associated cognitive dysfunction^2^. Interestingly, increased accumulation of amyloid β (Aβ) and p-tau in the brains of sucrose-treated mice (which have metabolic alterations resembling those of type 2 DM) has been reported, which suggests that (pre)diabetes might be a risk factor for AD^3^.

Erythropoietin (EPO) was originally identified as a cytokine responsible for inducing the production of red blood cells by blocking the apoptosis of progenitor cells^4^. EPO is a 30.5 kDa protein, and is produced by liver hepatocytes during the fetal period and primarily by the kidney after the birth^5^. EPO is also produced in central nervous system (CNS) tissue, and the EPO receptor (EPOR) homodimer is expressed in most CNS cell types, including neurons, astrocytes, and microglia^6^. EPO has attracted interest in recent years as a potential neuroprotective treatment in various neuropathologies. The neuroprotective effect of EPO is deemed to result from neurotrophic, anti-oxidant, antiapoptotic, angiogenic and anti-inflammatory effects of the hormone^4, 7–11^. It has been demonstrated that EPO possesses a neuroprotective function in animal models of traumatic brain injury (TBI)^12^ and Aβ-induced AD^13^. Moreover, in cell cultures, EPO is capable of protecting cells from apoptosis^14, 15^.

Once EPO binds to the EPOR, a ligand-induced conformational change in the receptor homodimer leads to the activation of Janus kinase-2 (JAK2), thereby providing docking sites for downstream SH2 domain-containing signaling molecules including STAT5, PI3K and Akt^16, 17^. While there is now ample evidence indicating that EPO is beneficial against various types of brain injury, little is known about the effects of EPO in diabetes-associated cognitive dysfunction. Therefore, the present study aimed to investigate whether EPO has protective effects in the context of diabetes-associated cognitive dysfunction *in vivo* and *in vitro*, and to identify the possible mechanism underlying these effects.

## Results

### The effects of glucose on cell viability

**Figure 1:**
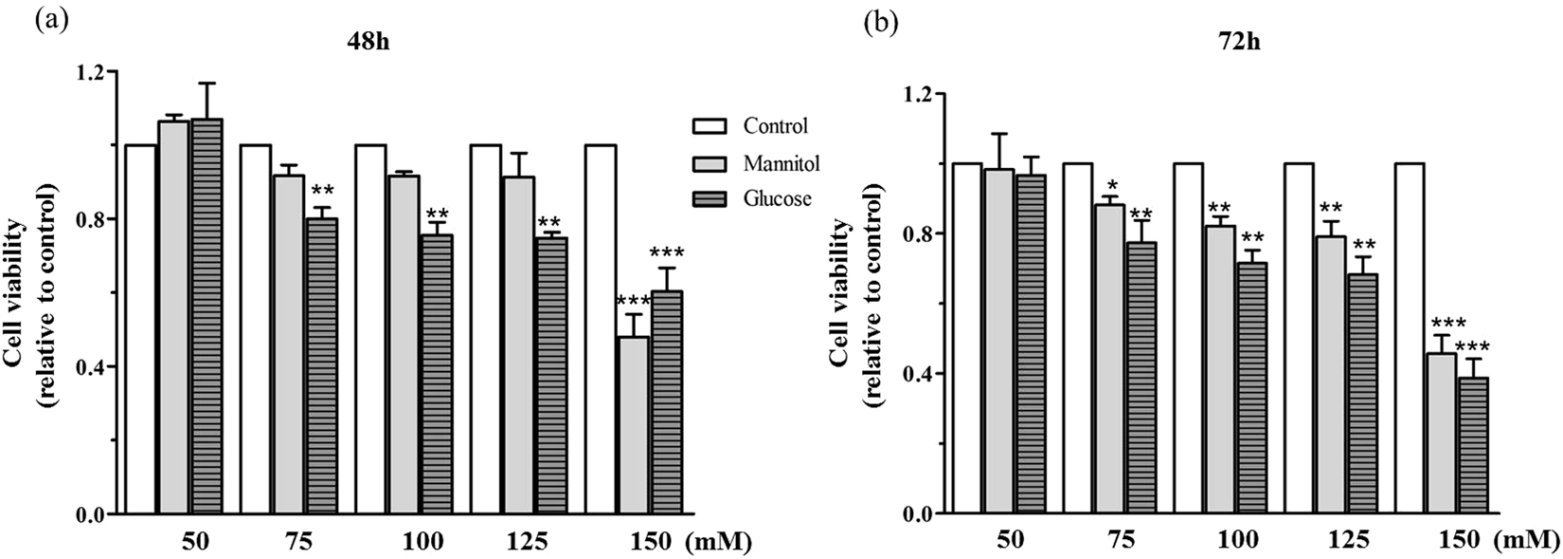
The effect of glucose and mannitol on cell viability. (**a**) The cell viability after 48 h glucose treatment. (**b**) The cell viability after 72 h glucose treatment. PC12 cells were treated with increasing concentrations of glucose or mannitol from 50–150 mM for 48 h and 72 h. Cell viability was measured by the MTT assay. The cell viability of each group was standardized to the control group. Data are expressed as the mean ± SEM, n = 4. *P < 0.05, **P < 0.01, and ***P < 0.001 vs. the control.

To investigate the influence of glucose on neuronal cell death, PC12 cells were exposed to increasing concentrations of glucose (from 50–150 mM) for 48 h and 72 h, and cell viability was then measured using a modified 3-(4,5-dimethylthiazol-2-yl)-2,5-diphenyl tetrazolium (MTT) assay. Cell cultures were exposed to equal concentrations of mannitol as a negative control for osmolarity. As shown in Fig. 1, the 48 h and 72 h treatment of PC12 cells with HG induced a concentration-dependent decrease in cell viability. When the value of the osmolarity increased to 150 mM, mannitol also could influence the cell viability. To exclude the factor of the osmolarity, HG (150 mM) should not be chosen. Since there was no significant difference in the cell viability between HG (100 mM) and HG (125 mM), treatment with of 100 mM glucose for 48 h was selected as the HG condition for further study.

### EPO inhibited HG-induced PC12 cell damage

**Figure 2:**
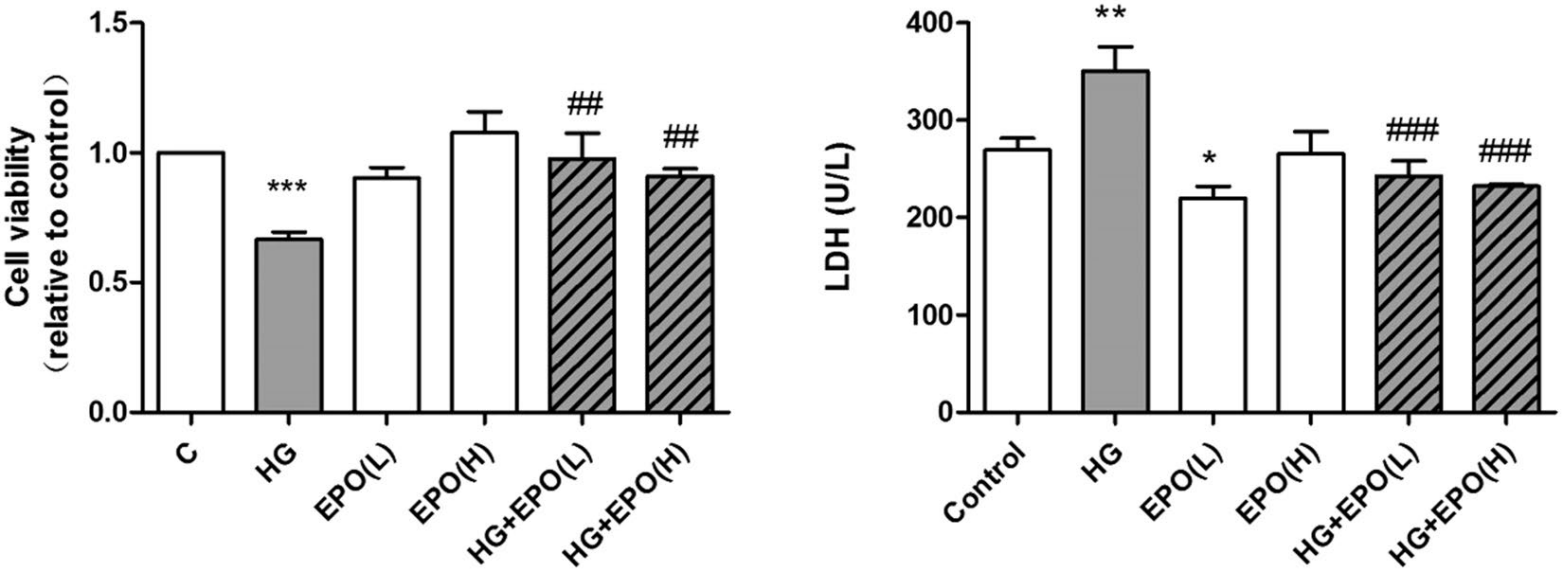
The effects of EPO on HG-induced PC12 cell damage. (**a**) The effects of EPO on cell viability. PC12 cells were treated with 100 mM glucose and various doses of EPO for 48 h. The cell viability was measured by the MTT assay. (**b**) The effects of EPO on HG-induced LDH release. After the treatments mentioned above, the LDH assay was carried out using an LDH-cytotoxicity colorimetric assay kit. Data are expressed as the mean ± SEM, n = 5. *P < 0.05, **P < 0.01, versus control. ^#^ *P* < 0.05, ^###^ *P* < 0.001, vs. the model. EPO (L), treatment with a low dose of EPO (8 U/mL); EPO (H), treatment with a high dose of EPO (16 U/mL); HG + EPO (L), treatment with 100 mM glucose and a low dose of EPO (8 U/mL); HG + EPO (L), treatment with 100 mM glucose and a high dose of EPO (16 U/mL).

The MTT assay and the LDH assay were used to examine whether EPO affects the extent of HG-induced PC12 cell damage. PC12 cells were treated with HG and different concentrations of EPO for 48 h. As shown in Fig. 2(a), the cell viability at 100 mM glucose was significantly lower than that of the control group (*P* < 0.01). Both doses of EPO increased viability. (The result of cell number is shown in the supplemental materials). Lactate dehydrogenase (LDH) leakage was used to assess necrotic cell death. As shown in Fig. 2(b), a significant increase in LDH release was detected in the HG group compared with the control group (*P* < 0.05). A rapid drop in LDH release was observed for each dose of EPO (*P* < 0.05).

### EPO ameliorated the oxidative stress induced by HG

**Figure 3:**
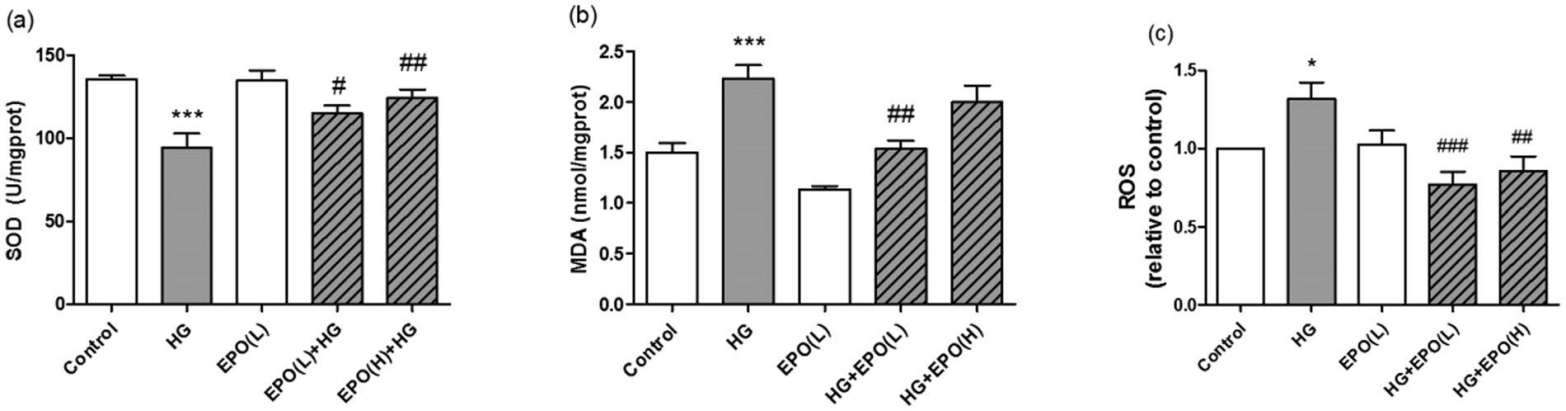
The effect of EPO on the levels of SOD (**a**), MDA (**b**) and ROS (**c**) in PC12 cells. PC12 cells were treated with 100 mM glucose and various doses of EPO for 48 h. DCFH-DA was used as a probe to evaluate intracellular ROS levels with a fluorescence microplate reader. The results are expressed as the mean ± SEM; n = 5. *P < 0.05 and ***P < 0.001 vs. the control treatment; ^#^ *P* < 0.05 and ^##^ *P* < 0.01 vs. HG treatment.

The quantification of superoxide dismutase (SOD), malondialdehyde (MDA) level and superoxide dismutase (ROS) production, as well as all statistically significant differences in these measurements, are shown in Fig. 3. SOD levels were significantly decreased (*P* < 0.001), while MDA and ROS levels were significantly increased in the HG condition in comparison to the control (*P* < 0.05). E*P*O increased the activity of SOD in the low- and high-dose group (*P* < 0.01). Additionally, the increases in MDA and ROS were markedly attenuated by the low dose of EPO.

### The protective effect of EPO against HG-induced apoptosis of PC12 cells involves the PI3K/Akt signaling pathway

**Figure 4:**
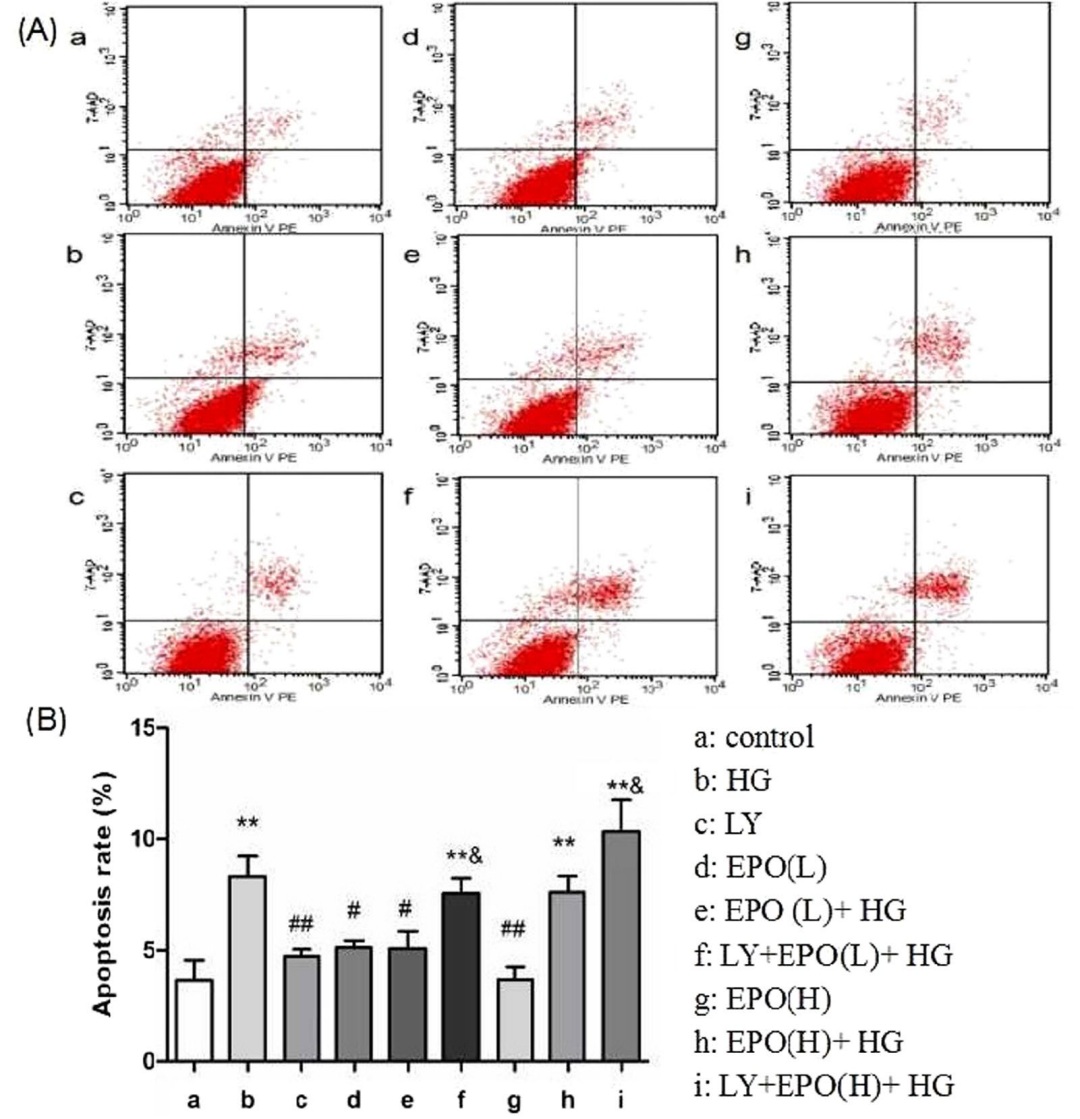
The effects of EPO on HG-induced apoptosis of PC12 cells in the absence or presence of LY. PC12 cells were treated with 100 mM glucose and various doses of EPO for 48 h in the absence or presence of 10 μM LY; if present, LY was added 2 h prior to HG and EPO treatment. Then, the PC12 cells were stained with annexin V-PE and 7-AAD, and the cells were analyzed by flow cytometry. (**A**) The extent of cell apoptosis, as determined by flow cytometry. (**B**) The apoptosis rates of PC12 cells receiving different treatments. Data are expressed as the mean ± SEM; n = 5. **P < 0.01 vs. the control treatment, ^#^ *P* < 0.05, ^##^ *P* < 0.01 vs. the HG treatment, and ^&^ *P* < 0.05 vs. the equal relative doses of EPO without LY.

To further evaluate whether the effects of EPO on HG-induced PC12 cell damage involves the PI3K/Akt signaling pathway, LY294002 (hereafter called LY), a PI3K inhibitor, was used. We investigated whether EPO influences the extent of cell apoptosis induced by HG. Cell apoptosis was quantified by flow cytometry using double staining with annexin V-PE and 7-AAD. As shown in Fig. 4(B), cells treated with 100 mM glucose exhibited a significantly increased apoptosis rate (*P* < 0.01) compared with the control cells. The low dose of EPO (8 U/mL) significantly decreased HG-induced PC12 cell apoptosis (*P* < 0.05). Treatment with LY (10 μM) alone did not affect PC12 cell apoptosis, but it abolished the protective effect of EPO on HG-treated PC12 cells (*P* < 0.05).

### EPO upregulated the expression of EPOR and regulated the PI3K/Akt/GSK-3β signaling pathway in PC12 cells

**Figure 5:**
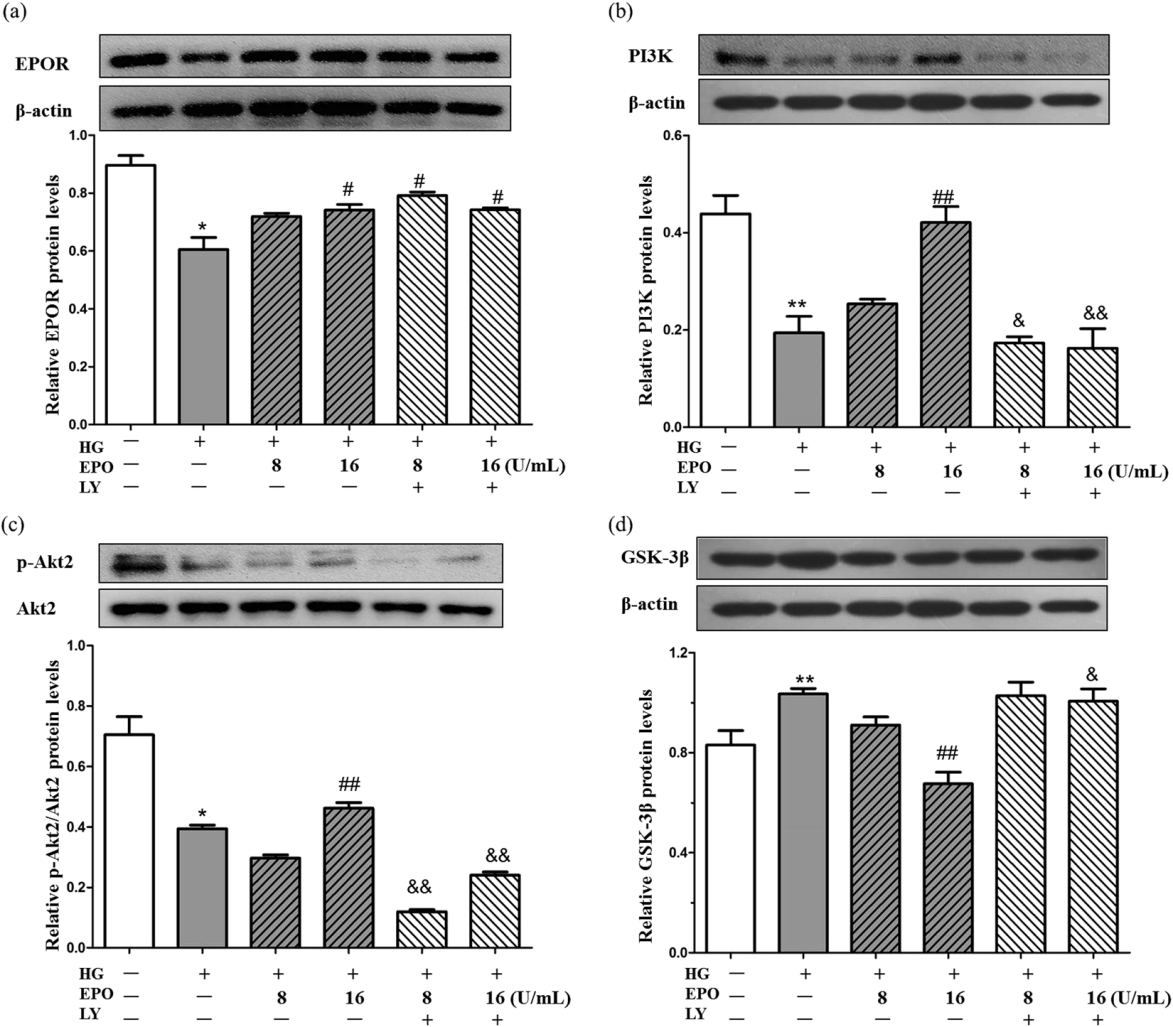
The involvement of the EPOR/PI3K/GSK-3β signaling pathway in the protective effects of EPO in PC12 cells. (**a**) The expression of EPOR protein in PC12 cells. (**b**) The expression of PI3K protein in PC12 cells. (**c**) The expression of p-Akt2 and the Akt2 protein in PC12 cells. (**d**) The expression of GSK-3β protein in PC12 cells. PC12 cells were treated with 100 mM glucose and various doses of EPO for 48 h in the absence or presence of 10 μM LY treatment; if present, LY was added 2 h prior to HG and EPO. Cell extracts were subjected to Western blotting. The results are shown as the mean ± SEM; n = 5. *P < 0.05, **P < 0.01 vs. the control group; ^#^ *P* < 0.05 and ^##^ *P* < 0.01 vs. the model group; ^&^ *P* < 0.05 and ^&&^ *P* < 0.01 vs. the equal relative doses of EPO without LY.

Although we had identified that the effects of EPO on HG-induced PC12 cell damage involve the PI3K/Akt signaling pathway, the mechanism by which EPO affects insulin signaling remained unclear. EPO-EPOR signaling is known to activate the PI3K/Akt signaling pathway, which regulates cell survival and proliferation. One of the effectors of the PI3K/Akt signaling pathway is GSK-3β, and the PI3K/Akt-induced inactivation of GSK-3β can result in the prevention or abatement of apoptotic injury in neurons^14^. As shown in Fig. 5, treatment with EPO (16 U/mL) stimulated a significant increase in EPOR protein levels (Fig. 5(a)) and PI3K levels (Fig. 5(b)). In addition, EPO enhanced the phosphorylation of Akt2 (Fig. 5(c)). Furthermore, treatment of cells with EPO decreased the expression of GSK-3β (Fig. 5(d)). (The mRNA expression of EPOR, PI3K and GSK-3β are shown in the supplemental materials).

To determine whether the PI3K/Akt/GSK-3β signaling pathway is involved in EPO-mediated protection, LY was used. We observed that LY thoroughly abolished the EPO-induced PI3K expression (Fig. 5(b)) as well as the EPO-induced phosphorylation of Akt2 (Fig. 5(c)). LY notably attenuated the EPO-induced downregulation of GSK-3β (Fig. 5(d)). These data indicate that the neuroprotective effect of EPO is dependent on the PI3K/Akt/GSK-3β signaling pathway.

### EPO prevents diabetes-associated spatial learning and memory deficits

**Figure 6:**
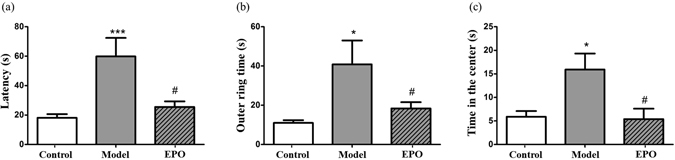
The effects of EPO on the learning and memory ability of diabetic rats in the Morris water maze test. (**a**) The effect of EPO on the escape latency of diabetic rats. The time spent in (**b**) the outer ring and (**c**) the center before finding the platform. The results are expressed as the mean ± SEM; n = 7. *P < 0.05, **P < 0.01 and ***P < 0.001 vs. the control; ^#^ *P* < 0.05 vs. the model.

The effect of EPO on diabetes-associated spatial learning and memory impairment was evaluated using the Morris water maze test. As shown in Fig. 6(a), the rats in the diabetic model group took markedly more time to find the platform than did the control group (*P* < 0.001), and the time that diabetic rats spent in the outer ring and center, which were far away from the platform, increased significantly compared with the control group (*P* < 0.05) (Fig. 6(b) and (c)). However, rats treated with EPO (300 U/kg) showed a markedly reduced the latency, outer ring time and central time compared with the rats in the diabetic model group (*P* < 0.05), suggesting that EPO could ameliorate the spatial learning and memory deficits in diabetic rats.

### EPO upregulated the expression of EPOR and regulated the PI3K/Akt/GSK-3β signaling pathway in the hippocampus

**Figure 7:**
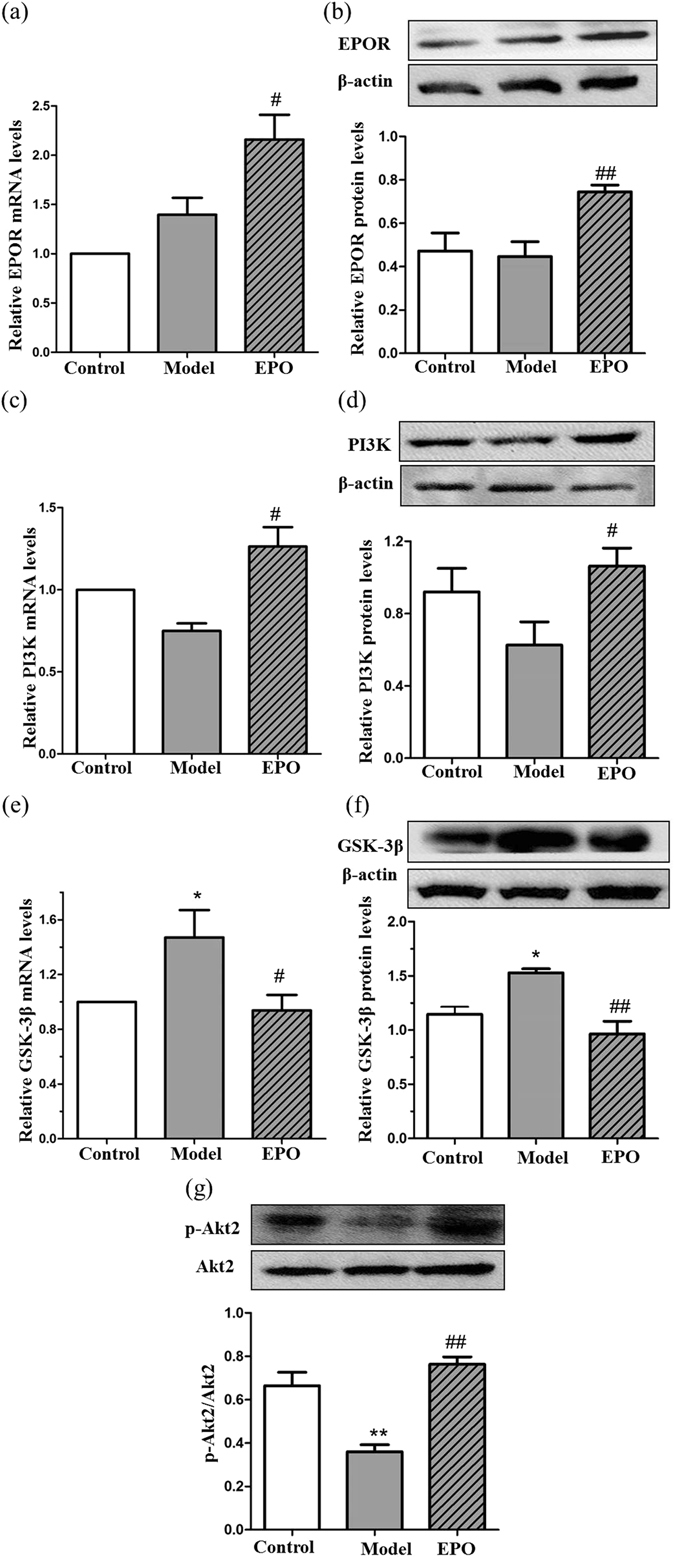
The involvement of EPOR/PI3K/GSK-3β in the protective effects of EPO in diabetic rats. (**a**) Hippocampal expression of EPOR mRNA. (**b**) Hippocampal expression of EPOR protein. (**c**) Hippocampal expression of PI3K mRNA. (**d**) Hippocampal expression of PI3K protein. (**e**) Hippocampal expression of GSK-3β mRNA. (**f**) Hippocampal expression of GSK-3β protein. (**g**) Hippocampal expression of p-Akt2 and the Akt2 protein. Gene expression was measured by real-time PCR. Relative mRNA abundance was measured in proportion to GAPDH. The results are shown as the mean ± SEM; n = 4. Protein expression levels in the lysates from freshly isolated hippocampus tissues were measured by Western blot analysis. The data are expressed as the mean ± SEM. *P < 0.05 and **P < 0.01 vs. the control group; ^#^ *P* < 0.05 and ^##^ *P* < 0.01 vs. the model group.

Then, we tested whether EPO played a role in the PI3K/Akt/GSK-3β signaling pathway in the experimental diabetic rats. As illustrated in Fig. 7, there is no significant difference between the model group and the control group in the relative mRNA and protein levels of EPOR (Fig. 7(a) and (b)) and PI3K (Fig. 7(c) and (d)) in the hippocampus. However, the model group exhibited increased relative mRNA levels (Fig. 7(e); *P* < 0.05) and protein levels (Fig. 7(f); *P* < 0.05) of GSK-3β, and decreased levels of p-Akt2/Akt2 (Fig. 7(g); *P* < 0.01) compared with the control group. Treatment with EPO (300 U/kg administered three times per week for 4 weeks) increased the mRNA-level (Fig. 7(a); *P* < 0.05) and protein-level (Fig. 7(b); *P* < 0.01) expression of EPOR, increased the mRNA-level (Fig. 7(c); P < 0.05) and protein-level (Fig. 7(d); *P* < 0.05) expression of PI3K, and significantly decreased relative mRNA levels (Fig. 7(e); *P* < 0.05) and protein levels (Fig. 7(f); *P* < 0.01) of GSK-3β. Moreover, E*P*O significantly increased the levels of p-Akt2/Akt2 in the hippocampi of diabetic rats (Fig. 7(g); *P* < 0.01).

## Discussion

EPO, a cytokine controlling red blood cell production, has various physiological functions, including neuroprotective, antiapoptotic, anti-inflammatory, and anti-oxidant effects^7–11^. Stivelman^18^ summarized that increase in hematocrit following EPO therapy can improve cognitive functions of haemodialysis patients. And improvement in uremic encephalopathy patients with anemia is well correlated with increase in hematocrit induced by EPO^19^. There are studies demonstrating that EPO exerts a neuroprotective function against brain ischemia-associated cognitive decline and TBI-induced cognitive deficits^20, 21^. We have previously shown that EPO improves glucose metabolism and mitigates pancreatic β-cell damage in the experimental diabetic rats^22^. However, the effects of EPO on diabetes-associated cognitive impairments and the mechanisms of such effects remained unknown. In this study, we evaluated the effect of EPO on diabetes-associated cognitive dysfunction using *in vitro* and *in vivo* models, and we then examined the mechanisms underlying the neuroprotective effects of EPO.

Oxidative stress plays a key role in the pathogenesis of type 2 DM, and increased oxidative stress can lead to neuronal injury. Oxidative changes amplify the production of ROS and trigger Aβ generation, tau phosphorylation and the formation of neurofibrillary tangles^23^. Excess formation of ROS is a common mechanism by which HG induces damage to neuronal cells, and causes nervous system injury and symptoms of diabetic neuropathy^24–26^. EPO has been shown to exert an anti-oxidant effect in type 1 and type 2 DM^9, 27^. Our results showed that EPO could decrease the HG-induced elevation of ROS production in PC12 cells. In addition, the current results indicated that HG raised the level of MDA, a biomarker of lipid peroxidation, and reduced the level of SOD. We found that EPO decreased the level of MDA and increased the activity of SOD in HG-treated PC12 cells. EPO may exert its anti-oxidant effects by modifying the activity of autophagy and thus limit neonatal brain damage^28^. The administration of EPO can also block apoptotic cell death during neuronal kainite-induced oxidative stress and vascular oxygenglucose deprivation^29^. Furthermore, our previous study suggested that EPO enhanced anti-oxidant activity in diabetic rats^22^.

Apoptotic cell death contributes significantly to the pathogenesis of cognitive dysfunction. Caspase-dependent apoptosis has been implicated in synaptic dysfunction/degeneration and neuronal cell loss in TBI and AD^30^. Caspase-3 activation is one of the main characteristics of many neurodegenerative diseases, including diabetes-associated cognitive deficits and Parkinson’s disease^31, 32^. In the present study, PC12 cells treated with 100 mM glucose exhibited an increased apoptosis rate compared with the control group. These data suggested that HG may increase caspase-3 expression levels and induce oxidative stress, leading to apoptosis. However, treatment with 8 U/ml EPO significantly decreased cell apoptosis. This result may be associated with an EPO-induced decrease in the levels of caspase-3 and oxidative stress.

Animal models have demonstrated that EPO promotes neuroprotection through EPOR under conditions of brain damage^33^. Once EPO is bound to EPOR, EPO generates intracellular signals by phosphorylating and activating JAK2. PI3K is one of the major downstream effectors of JAK2^34^. Signore *et al*. reported that the activation of the PI3K-mediated pathway is sufficient to attenuate 6-OHDA toxicity in MN9D cells^15^. Studies by Ma *et al*. demonstrated that the activation of PI3K and its downstream target Akt by EPO plays an important role in inhibiting apoptosis in PC12 cells exposed to β-amyloid 25–35^14^. Our results also suggest that the PI3K/Akt pathway is activated by EPO. There was a significant increase in the protein-level expression of EPOR, PI3K and Akt2 protein in PC12 cells and in diabetic rats exposed to EPO. GSK-3β, a downstream target of PI3K/Akt, has been shown to be involved in the hyperphosphorylation of tau and the neurotoxicity of Aβ in AD^35^. In agreement with a previous report showing that GSK-3β activity is suppressed by EPO^36^, our data indicated that EPO was able to enhance the level of the inactive form of GSK-3β in parallel with the activation of Akt. Meanwhile, EPO upregulated the expression of EPOR and PI3K, and downregulated the expression of GSK-3β in the diabetic rats.

**Figure 8:**
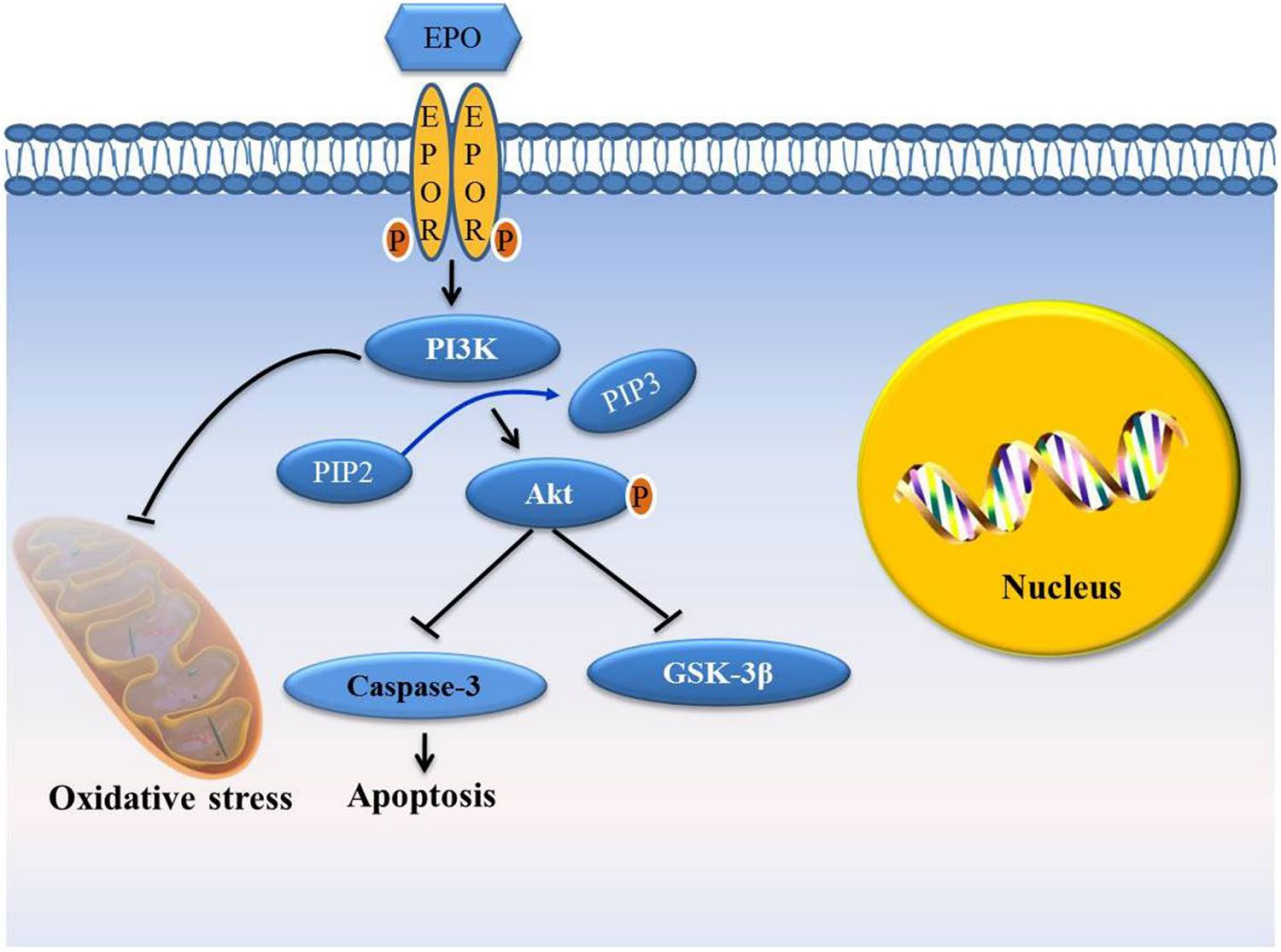
A diagram illustrating possible signaling pathways involved in EPO-mediated neuroprotection. EPO binds to EPOR and induces receptor dimerization, which leads to the activation of PI3K. Activated PI3K increases the phosphorylation of Akt2. p-Akt2 can inhibit the expression of GSK-3β, which is involved in the hyperphosphorylation of tau and the neurotoxicity of Aβ in AD. In addition, p-Akt2 is involved in the inhibition of cell apoptosis. Moreover, activated PI3K may play a vital role in ameliorating oxidative stress.

To further investigate the role of PI3K/Akt/GSK-3β in diabetes-associated cognitive impairments, we used LY. LY is known as a specific inhibitor of the PI3K/Akt kinase pathway. In a study by Zhu *et al*.^37^, LY was used to block the activation of the PI3K pathway to examine whether isoquercitrin induced the inactivation of GSK3β by activating the PI3K/Akt pathway. We found that pretreatment with LY resulted in a significant decrease in the protein-level expression of PI3K and Akt2, and an increase in that of GSK-3β, compared with EPO alone. On the other hand, the protective effects of EPO against cell apoptosis were reserved by LY. These findings suggest that the inhibition of PI3K activity completely abolished the protective effects of EPO (Fig. 8). In addition to PI3K/Akt/GSK-3β signaling, there are many other EPO-induced intracellular signaling pathways through JAK2 activate multiple downstream signaling molecules including STAT5, PI3K/Akt, NF-κB, and MAPK^38^. EPO might exert anti-apoptotic action through other pathway simultaneously.

In conclusion, our data highlight the activation of the PI3K/Akt/GSK-3β signaling pathway as a potential mechanism by which EPO protects neurons from diabetic lesions. We found that EPO caused a downregulation of GSK-3β and an upregulation of EPOR, PI3K and p-Akt2/Akt2, and eventually led to improvements in the learning and memory deficits of diabetic rats, cell damage and oxidative stress. In addition, the protective effects of EPO were abolished by LY, a specific inhibitor of the PI3K/Akt kinase pathway. Overall, the data revealed that EPO could improve neuronal cell viability, LDH release and oxidative stress in PC12 cells, and could increase learning and memory capacity in diabetic rats via EPOR and the PI3K/Akt2/GSK-3β signaling pathway.

## Methods

### Cell culture

Rat pheochromocytoma PC12 cells were obtained from the Institute of Neurobiology (Xi’an Jiaotong University, Xi’an, Shaanxi, China) and grown in Dulbecco’s modified Eagle’s medium (DMEM, HyClone, USA) with 10% (v/v) heat-inactivated fetal bovine serum (HyClone, USA), and 1% (v/v) penicillin-streptomycin (100 U/ml penicillin and 100 μg/ml streptomycin, Solarbio, Beijing, China). The cells were maintained at 37 °C in a 90% humidified atmosphere containing 5% CO_2_. The concentration of glucose in the DMEM was 25 mM.

### Cell viability assay

Cell viability was determined using the MTT assay. Cell cultures that were exposed to equal concentrations of mannitol were used as osmolar controls. After two passages, PC12 cells were plated at a density of 5000 per well in a 96-microplate well and exposed to HG after 24 h. Cells were cultured in DMEM containing 25 mM (normal glucose control), 50 mM, 75 mM, 100 mM, 125 mM, and 150 mM glucose (as HG conditions) for 24, 48 and 72 h to determine the optimal HG concentration and culture duration. Then, 20 μl MTT (Sigma-Aldrich, USA) was added, and the cells were incubated for 4 h at 37 °C. The medium was then removed, and 150 μl DMSO (Sigma-Aldrich, USA) was added. After the cells and dye crystals were solubilized, the absorption was measured at 490 nm by an ELISA reader (BioTek, USA). Three independent experiments were performed for the experimental condition. For EPO treatment, cells were exposed to EPO at concentrations of 8 U/mL and 16 U/mL to examine the effective concentration.

### LDH assay

Lactate dehydrogenase (LDH), as a cytoplasmic enzyme, is not normally released from cells, but leaks out when the cell membrane’s integrity is compromised. The LDH test is a colorimetric assay for the quantification of cell death. An LDH assay was carried out using a BioVision LDH-cytotoxicity colorimetric assay kit (Nanjing Jiancheng Biology Engineering Institute, Nanjing, China) as per the manufacturer’s instructions. The optical density of the samples was measured at 490 nm by an ELISA reader (BioTek, USA).

### Apoptosis analysis

Apoptosis rates were quantified by flow cytometry with an annexin V-PE/7-AAD assay kit (BD Bioscience, Bedford, MA, USA) according to the manufacturer’s recommended procedure. Cells were seeded in 6-well culture plates. After EPO and HG (100 mM) incubation in the presence or absence of LY (Sigma-Aldrich, USA) for 48 h, the cells were collected and rinsed with PBS (2 × 5 min). Then, the cells were suspended in 1 mL binding buffer at a concentration of 1 × 10^6^ cells/mL. One hundred microliters of the cell solution was transferred to a 5 mL tube, and 5 μL of PE annexin V and 5 μL of 7-AAD were added. The cells were gently vortexed and then incubated for 15 min at RT in the dark. Four hundred microliters of binding buffer was added to each tube. The cells were analyzed using a flow cytometer (BD Bioscience, USA).

### Biochemical analysis

The levels of SOD and MDA were measured using a total superoxide dismutase assay kit (hydroxylamine method) and a malondialdehyde assay kit (TBA method) according to the manufacturer’s instructions (Nanjing Jiancheng Bioengineering Institute, Nanjing, China).

### ROS detection

Cells (1 × 10^5^) were seeded in 6-well plates and incubated for 48 h before any treatment. ROS generation after the treatment of EPO was evaluated using 2,7-dichlorofluorescin diacetate (DCFH-DA; Nanjing Jiancheng Biology Engineering Institute, Nanjing, China). After all treatments, the cells were incubated with 10 μM DCFH-DA for 30 min at 37 °C. ROS levels were quantified using a fluorescence microplate reader (PerkinElmer Inc., Waltham, MA, USA).

### Animals, grouping and treatment

Thirty male SD rats weighing 160–180 g (supplied by the Experimental Animal Center of Xi’an Jiaotong University, China), were used in accordance with recommended guidelines on the care and use of laboratory animals issued by the Chinese Council on Animal Research. The study was subject to approval by the ethics committee at Xi’an Jiaotong University. All rats were housed in a temperature-controlled room with a 12 h light/12 h dark cycle and had free access to food and water.

After one week of acclimation, the rats were randomly divided into 2 groups: control (n = 8) and diabetic model (n = 22). The control rats were fed a standard laboratory diet (carbohydrates: 30%; proteins: 22%; lipids: 12%; vitamins: 3%). The diabetic rats were fed on a high-fat, HG diet (ingredients: 10% refined lard, 20% sucrose, 2.5% cholesterol, 1% sodium cholate and 66.5% common food), which was provided by the Laboratory Animal Center of Xi’an Jiaotong University. Six weeks later, the model rats were given a peritoneal injection of freshly prepared streptozotocin (STZ; 25 mg/kg body weight; Sigma, St Louis, USA) in 0.1 M citrate buffer (pH 4.5) after fasting overnight, while the control group was given an equivalent volume of citric acid buffer. Four weeks after STZ injection, fasting blood glucose (FBG) levels were measured, and rats with a FBG concentration of >16.7 mmol/L were considered diabetic (n = 16).

The diabetic rats were again randomly divided into a diabetic model group (model; n = 8) and an EPO-treated group (EPO; n = 8). Recombinant human erythropoietin (rHuEPO; Shenyang Sunshine Pharmaceutical CO. LTD., Shenyang, China) was administered to the diabetic rats by hypodermic injection at 300 U/kg 3 times a week for 4 weeks. The equivalent volume of normal saline was administered to the control rats and the model rats.

### Morris water maze test

Subsequently, the water maze test was performed to examine spatial memory. The maze consisted of a circular pool that was approximately 2.14 m in diameter and 0.4 m deep, and was filled to a depth of 0.25 m with water that had a temperature of 21 ± 1 °C. A circular platform was submerged 1 cm below the water level in the middle of the pool. Pretraining began one day prior to the formal test in order for the rats to adapt to the room, experimenter, and water. The rats were tested for five days, with four trials per day. The location of the platform remained the same throughout the whole experiment. For all training trials, the rats were positioned in a random starting location in different quadrants, and they faced the edge of the pool. The rats were given 60 s to swim freely to find the hidden platform and remain on it for 15 s. The time spent on reaching the platform was recorded. If a rat failed to locate the platform within 60 s, the experimenter guided the subject to the platform and allowed it to remain there for 15 s, and the escape latency was recorded as 60 s^39, 40^.

### Real time-PCR

Total RNA was isolated from rat hippocampus tissue and from cells using the TriPure RNA isolation reagent. Four micrograms of RNA was reverse transcribed using the Prime Script RT Master Mix (Perfect Real-Time; TaKaRa Bio, Inc., Tokyo, Japan) according to the manufacturer’s protocol. Quantitative real-time PCR was performed using SYBR Premix Ex Taq II (Perfect Real-Time; TaKaRa Bio, Inc., Tokyo, Japan). The PCR reactions were performed in 96-well plates in an iQ5 Real-Time PCR Detection System (Bio-Rad Laboratories, Hercules, CA). GAPDH served as an endogenous control, and the expression levels of the other tested genes were normalized to the level of GAPDH. The standard curve was produced and the data analyzed using Bio-Rad iQ5 software (Bio-Rad Laboratories, Hercules, CA).

The primers included were the following: EPOR forward 5′-TCT CAC TGT TGC TGA CTG TGC TG-3′; EPOR reverse 5′-AAG TTA CCC TTG TGG GTG AA-3′; PI3K forward 5′-GGC TGC TAT GCC TGC TCT GTA-3′; PI3K reverse 5′-CAT AGC CGG TGG CAG TCT TG-3′; GSK-3β forward 5′-TTC TCG GTA CTA CAG GGC ACC AG-3′; GSK-3β reverse 5′-GTC CTA GCA ACA ATT CAG CCA ACA-3′; GAPDH forward 5′-GAC AAC TTT GGC ATC GTG GA-3′; and GAPDH reverse 5′-ATG CAG GGA TGA TGT TCT GG-3′.

### Western blot analysis

Western blotting was conducted using the standard method. Briefly, PC12 cells and hippocampus tissue were lysed in radioimmunoprecipitation assay (RIPA; Pioneer Biotechnology, Xi’an, China) lysis buffer containing the Halt protease inhibitor cocktail (Roche, Basel, Switzerland). After incubation on ice for 30 min, lysates were clarified by centrifugation at 12,000 g for 10 min at 4 °C. For hippocampus tissue, 50 mg tissue was ground manually in 0.5 mL RIPA lysate containing the Halt protease inhibitor cocktail. We obtained the supernatant by centrifuging the samples at 12000 g for 10 min at 4 °C and determined the protein concentration of the supernatant with a bicinchoninic acid (BCA; Heart, Xi’an, China) protein assay kit. Then, 80 μg of total protein from each sample was subjected to SDS-polyacrylamide gel electrophoresis (PAGE) (10%), and the proteins were transferred to a PVDF membrane (Millipore, MA, USA). Then, we blocked the membranes with 5% nonfat milk, and incubated them with primary antibodies against phospho-Akt (1:1000; Cell Signaling Technology, CST, Boston, USA), GSK-3β (1:5000; Cell Signaling Technology, CST, Boston, USA), Akt (1:1000; Abcam, Cambridge, UK), PI3K (1:1000; Abcam, Cambridge, UK), EPOR (1:500; Abcam, Cambridge, UK), and β-actin (Santa Cruz Biotechnology, Santa Cruz, CA, USA) at 4 °C overnight. The membranes were subsequently washed thrice with Tris-buffered saline containing TWEEN 20 (20 mM Tris, pH 7.5, 150 mM NaCl, 0.1% TWEEN 20; TBST) and probed with the corresponding secondary antibodies conjugated with HRP at room temperature for 1 h. The immunoreactive bands were visualized with an ECL Western blot kit (Thermo, MA, USA) according to the manufacturer’s instructions.

### Statistical analysis

Data were expressed as the mean ± SEM from three independent experiments. Two group comparisons were evaluated by independent *t*-tests, as appropriate. Multiple comparisons were analyzed by ANOVA followed by Fisher’s least significant difference test (LSD-t), and were performed using SPSS v18.0 software (SPSS, Inc., Shanghai, China). All *P* values were two-sided, and a value of *P* < 0.05 was considered statistically significant.

## Electronic supplementary material

Supplementary information

## References

[1] Duarte AI (2013). Crosstalk between diabetes and brain: Glucagon-like peptide-1 mimetics as a promising therapy against neurodegeneration. BBA-Mol Basis Dis.

[2] Pasquier F, Boulogne A, Leys D, Fontaine P (2006). Diabetes mellitus and dementia. Diabetes Metab.

[3] Carvalho C (2012). Metabolic alterations induced by sucrose intake and Alzheimer’s disease promote similar brain mitochondrial abnormalities. Diabetes.

[4] Bond WS, Rex TS (2014). Evidence That Erythropoietin Modulates Neuroinflammation through Differential Action on Neurons, Astrocytes, and Microglia. Front Immunol.

[5] Erbaş O, Çınar BP, Solmaz V, Çavuşoğlu T, Ateş U (2015). The neuroprotective effect of erythropoietin on experimental Parkinson model in rats. Neuropeptides.

[6] Genc S, Koroglu TF, Genc K (2004). Erythropoietin and the nervous system. Brain Res.

[7] Nairz M, Sonnweber T, Schroll A, Theurl I, Weiss G (2012). The pleiotropic effects of erythropoietin in infection and inflammation. Microbes Infect.

[8] Wang L, Di L, Noguchi CT (2014). Erythropoietin, a novel versatile player regulating energy metabolism beyond the erythroid system. Int J Biol Sci.

[9] Zhang Y (2014). Erythropoietin action in stress response, tissue maintenance and metabolism. Int J Mol Sci.

[10] Stoyanoff TR, Todaro JS, Aguirre MV, Zimmermann MC, Brandan NC (2014). Amelioration of lipopolysaccharide-induced acute kidney injury by erythropoietin: involvement of mitochondria-regulated apoptosis. Toxicology.

[11] Subiros N, Del BD, Coro-Antich RM (2012). Erythropoietin: still on the neuroprotection road. Ther Adv Neurol Disord.

[12] Hellewell SC, Yan EB, Alwis DS, Bye N, Morganti-Kossmann MC (2013). Erythropoietin improves motor and cognitive deficit, axonal pathology, and neuroinflammation in a combined model of diffuse traumatic brain injury and hypoxia, in association with upregulation of the erythropoietin receptor. J Neuroinflammation.

[13] Esmaeili Tazangi P, Moosavi SMS, Shabani M, Haghani M (2015). Erythropoietin improves synaptic plasticity and memory deficits by decrease of the neurotransmitter release probability in the rat model of Alzheimer’s disease. Pharmacol Biochem Be.

[14] Ma R (2009). Erythropoietin protects PC12 cells from β-amyloid(25–35)-induced apoptosis via PI3K/Akt signaling pathway. Neuropharmacology.

[15] Signore AP (2006). Erythropoietin protects against 6-hydroxydopamine-induced dopaminergic cell death. J Neurochem.

[16] Ma R (2014). JAK2/STAT5/Bcl-xL signalling is essential for erythropoietin-mediated protection against apoptosis induced in PC12 cells by the amyloid beta-peptide Abeta25–35. Br J Pharmacol.

[17] Alexander SP (2013). The Concise Guide to PHARMACOLOGY 2013/14: enzymes. Br J Pharmacol.

[18] Stivelman JC (2000). Benefits of anaemia treatment on cognitive function. Nephrol Dial Transplant.

[19] Nissenson AR (1992). Epoetin and cognitive function. Am J Kidney Dis.

[20] Hralova M (2014). Effects of a single dose of erythropoietin on motor function and cognition after focal brain ischemia in adult rats. Prague Med Rep.

[21] Schober ME (2014). Erythropoietin improved cognitive function and decreased hippocampal caspase activity in rat pups after traumatic brain injury. J Neurotrauma.

[22] Chen LN (2015). Erythropoietin improves glucose metabolism and pancreatic beta-cell damage in experimental diabetic rats. Mol Med Rep.

[23] Kawser Akter EALS (2010). Diabetes mellitus and Alzheimer’s disease: shared pathology and treatment?. Brit J Clin Pharmaco.

[24] Aminzadeh A, Dehpour AR, Safa M, Mirzamohammadi S, Sharifi AM (2014). Investigating the protective effect of lithium against high glucose-induced neurotoxicity in PC12 cells: involvements of ROS, JNK and P38 MAPKs, and apoptotic mitochondria pathway. Cell Mol Neurobiol.

[25] Shahveisi K (2014). The role of local renin-angiotensin system on high glucose-induced cell toxicity, apoptosis and reactive oxygen species production in PC12 cells. Iran J Basic Med Sci.

[26] Renaud J, Bournival J, Zottig X, Martinoli MG (2014). Resveratrol protects DAergic PC12 cells from high glucose-induced oxidative stress and apoptosis: effect on p53 and GRP75 localization. Neurotox Res.

[27] Yu T (2014). Erythropoietin attenuates oxidative stress and apoptosis in Schwann cells isolated from streptozotocin-induced diabetic rats. J Pharm Pharmacol.

[28] Bendix I (2012). Erythropoietin modulates autophagy signaling in the developing rat brain in an *in vivo* model of oxygen-toxicity. Int J Mol Sci.

[29] Maiese K (2015). Erythropoietin and diabetes mellitus. World J Diabetes.

[30] Walker KR, Tesco G (2013). Molecular mechanisms of cognitive dysfunction following traumatic brain injury. Front Aging Neurosci.

[31] Sadeghi A, Hami J, Razavi S, Esfandiary E, Hejazi Z (2016). The Effect of Diabetes Mellitus on Apoptosis in Hippocampus: Cellular and Molecular Aspects. Int J Prev Med.

[32] Zawada WM (2015). Loss of angiotensin II receptor expression in dopamine neurons in Parkinson’s disease correlates with pathological progression and is accompanied by increases in Nox4- and 8-OH guanosine-related nucleic acid oxidation and caspase-3 activation. Acta Neuropathol Commun.

[33] Castaneda-Arellano R, Feria-Velasco AI, Rivera-Cervantes MC (2014). Activity increase in EpoR and Epo expression by intranasal recombinant human erythropoietin (rhEpo) administration in ischemic hippocampi of adult rats. Neurosci Lett.

[34] Zhao W, Kitidis C, Fleming MD, Lodish HF, Ghaffari S (2006). Erythropoietin stimulates phosphorylation and activation of GATA-1 via the PI3-kinase/AKT signaling pathway. Blood.

[35] Maurice T (2013). Intranasal formulation of erythropoietin (EPO) showed potent protective activity against amyloid toxicity in the Aβ_25-35_ non-transgenic mouse model of Alzheimer’s disease. J Psychopharmacol.

[36] Somervaille TC, Linch DC, Khwaja A (2001). Growth factor withdrawal from primary human erythroid progenitors induces apoptosis through a pathway involving glycogen synthase kinase-3 and Bax. Blood.

[37] Zhu M (2016). Isoquercitrin Inhibits Hydrogen Peroxide-Induced Apoptosis of EA.hy926 Cells via the PI3K/Akt/GSK3beta Signaling Pathway. Molecules.

[38] Ma, C. *et al*. Erythropoietin Pathway: A Potential Target for the Treatment of Depression. *Int J Mol Sci* **17**, pii: E677 (2016). 10.3390/ijms17050677 PMC4881503 27164096

[39] Lowry NC, Pardon LP, Yates MA, Juraska JM (2010). Effects of long-term treatment with 17 β-estradiol and medroxyprogesterone acetate on water maze performance in middle aged female rats. Horm Behav.

[40] Zhang G (2015). Wnt-5a prevents Aβ-induced deficits in long-term potentiation and spatial memory in rats. Physiol Behav.

