# Supplementary material for: Erythropoietin ameliorates diabetes-associated cognitive dysfunction in vitro and in vivo
Source: Sci Rep. 2017 Jun 5;7:2801. doi: 10.1038/s41598-017-03137-6 (PMC5459814; doi:10.1038/s41598-017-03137-6)

Title page

Title: Erythropoietin ameliorates diabetes-associated cognitive dysfunction *in vitro* and *in vivo*

Short title: Erythropoietin ameliorates diabetes-associated cognitive dysfunction

Authors: Meng Wang [a, c 1], Wenhui Yan [a 1], Yuan Liu [a], Hao Hu [a], Qiang Sun [a], Xinlin Chen [d], Weijin Zang [a, b] & Lina Chen [a, b *]

Affiliations:

[a] Department of Pharmacology, School of Basic Medical Sciences, Xi'an Jiaotong University Health Science Center, Xi'an 710061, Shaanxi, China

[b] Key Laboratory of Environment and Genes Related to Diseases (Xi'an Jiaotong University), Ministry of Education, Xi'an 710061, Shaanxi, China

[c] Shijiazhuang Obstetrics and Gynecology Hospital, Shijiazhuang 050000, Hebei, China

[d] Institute of Neurobiology, School of Basic Medical Sciences, Xi'an Jiaotong University Health Science Center, Xi'an 710061, Shaanxi, China

[1] These authors contributed equally to this manuscript.

*** Corresponding author:**

Lina Chen

Tel.: +86-18220554189

Fax: +86-29-82655032

E-mail address: chenlin@mail.xjtu.edu.cn (L. Chen)

Supplementary information

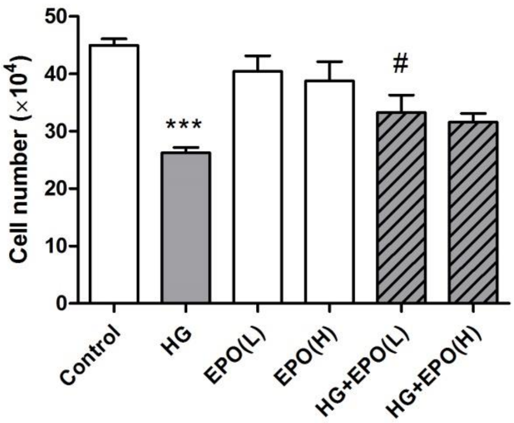

Figure 1 (supplement). The cell numbers of EPO on high glucose HG-induced PC12 cell damage.

PC12 cells were treated with 100 mM glucose and various doses of EPO for 48 h. The cell numbers were observed with cell counting chamber under microscope. Data are expressed as the mean ± SEM, n=5. $^{***}P$<0.001, versus control. $^{\#}P$<0.05, vs. the model. EPO (L), treatment with a low dose of EPO (8 U/mL); EPO (H), treatment with a high dose of EPO (16 U/mL); HG+EPO (L), treatment with 100 mM glucose and a low dose of EPO (8 U/mL); HG+EPO (L), treatment with 100 mM glucose and a high dose of EPO (16 U/mL).

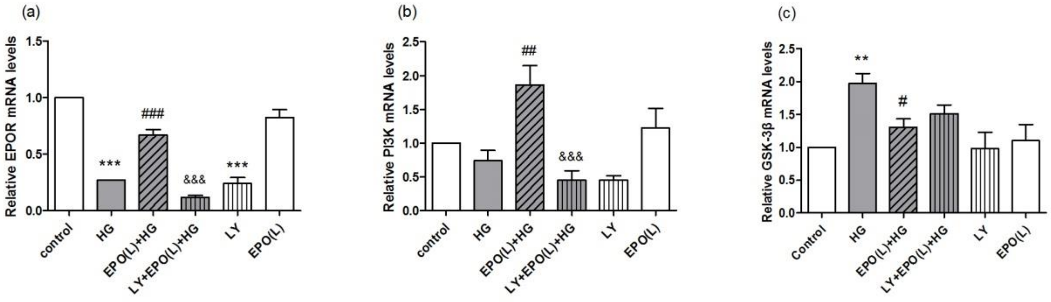

Figure 2 (supplement). The mRNA expression of EPOR (a), PI3K (b), and GSK3β (c) of EPO in HG-induced PC12 cells.

(a) The mRNA expression of EPOR in PC12 cells. (b) The mRNA expression of PI3K in PC12 cells. (c) The mRNA expression of GSK-3βin PC12 cells. PC12 cells were treated with 100 mM glucose and various doses of EPO for 48 h in the absence

or presence of 10 μM LY treatment; if present, LY was added 2 h prior to HG and EPO. Cell extracts were subjected to real time-PCR. The results are shown as the mean ± SEM; n=3. $^{**}P$<0.01, and $^{***}P$<0.001 vs. the control group; $^{\#}P$<0.05, $^{\#\#}P$<0.01, and $^{\#\#\#}P$<0.001 vs. the model group; $^{\&\&\&}P$<0.001 vs. the equal relative doses of EPO without LY.

Western blots of figure 5:

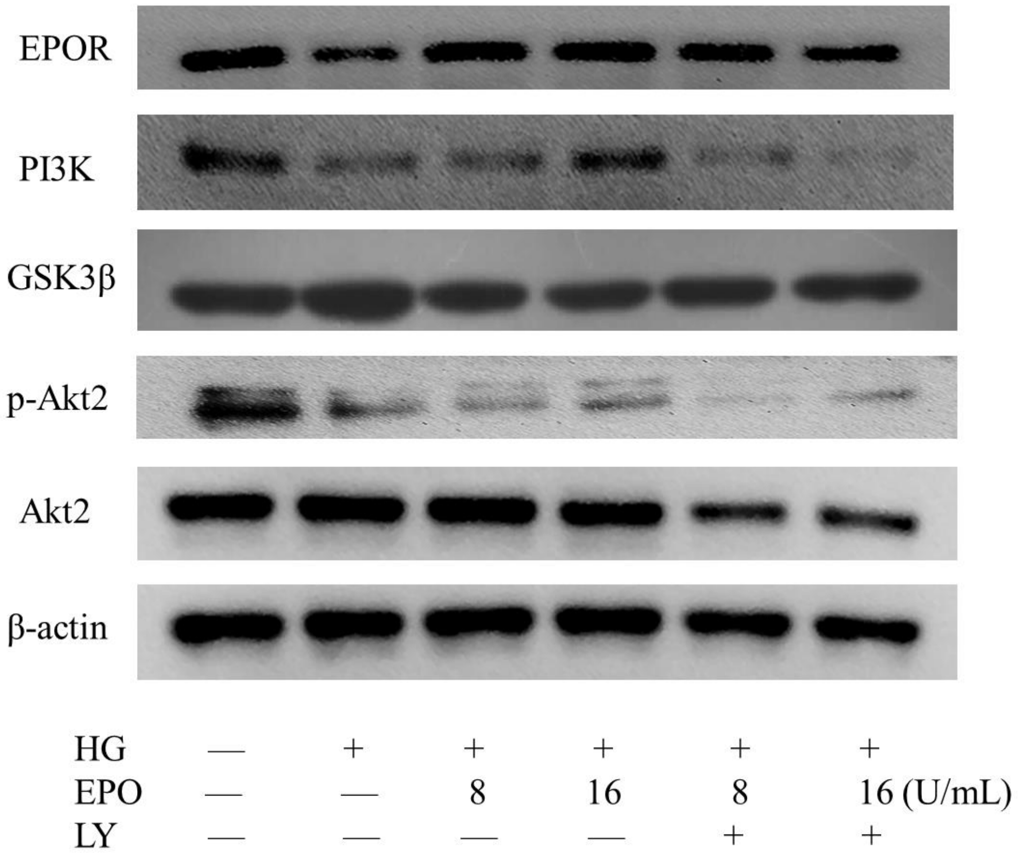

Western blots of figure 7:

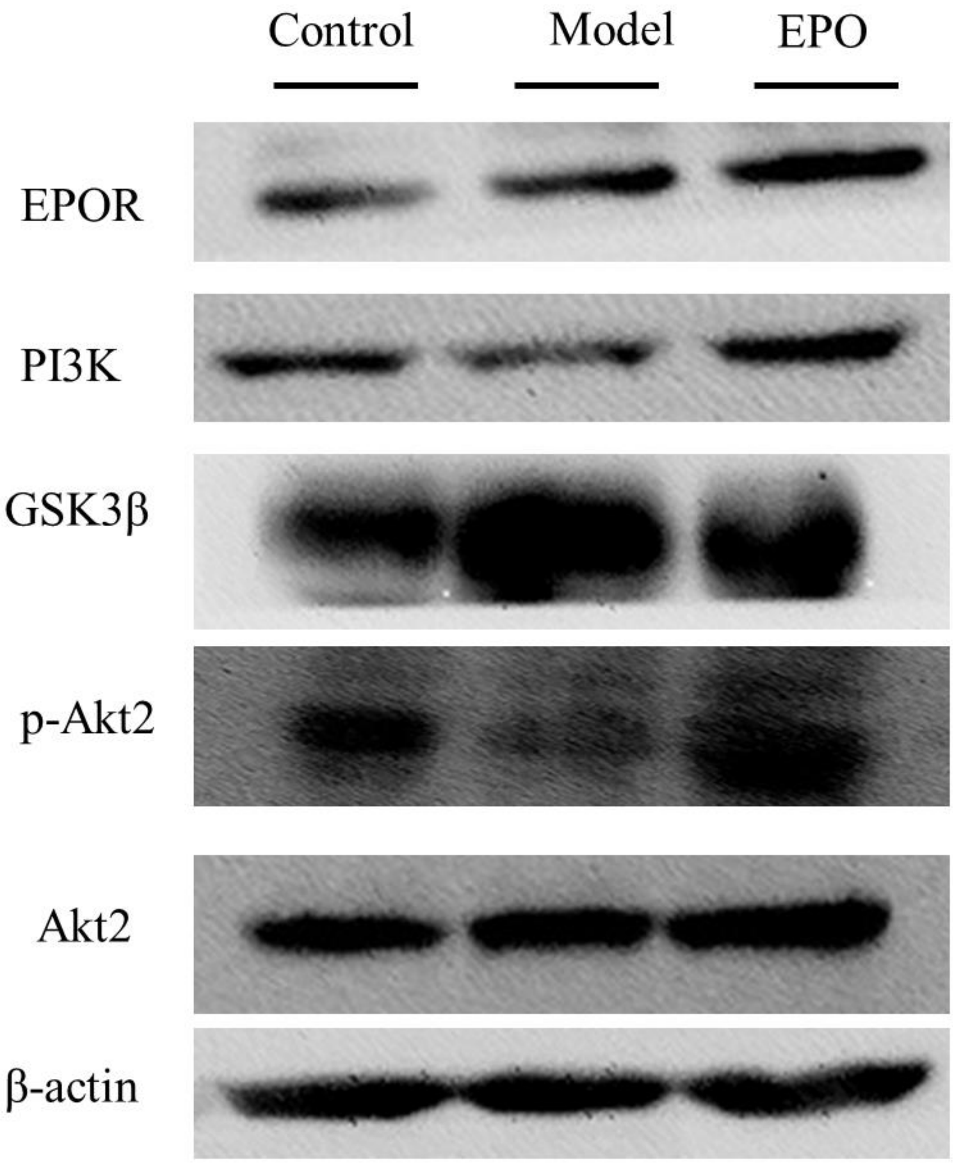

Supplement: Supplementary file 1 — Supplementary information [file 41598_2017_3137_MOESM1_ESM.pdf]
